# Microfluidics-Based POCT for SARS-CoV-2 Diagnostics

**DOI:** 10.3390/mi13081238

**Published:** 2022-08-01

**Authors:** Binfeng Yin, Xinhua Wan, A. S. M. Muhtasim Fuad Sohan, Xiaodong Lin

**Affiliations:** 1 School of Mechanical Engineering, Yangzhou University, Yangzhou 225127, China; mx120210515@stu.yzu.edu.cn (X.W.); 188801007@yzu.edu.cn (A.S.M.M.F.S.); 2 College of Food and Biological Engineering, Zhengzhou University of Light Industry, Zhengzhou 450001, China

**Keywords:** microfluidic, SARS-CoV-2, point of care testing

## Abstract

A microfluidic chip is a tiny reactor that can confine and flow a specific amount of fluid into channels of tens to thousands of microns as needed and can precisely control fluid flow, pressure, temperature, etc. Point-of-care testing (POCT) requires small equipment, has short testing cycles, and controls the process, allowing single or multiple laboratory facilities to simultaneously analyze biological samples and diagnose infectious diseases. In general, rapid detection and stage assessment of viral epidemics are essential to overcome pandemic situations and diagnose promptly. Therefore, combining microfluidic devices with POCT improves detection efficiency and convenience for viral disease SARS-CoV-2. At the same time, the POCT of microfluidic chips increases user accessibility, improves accuracy and sensitivity, shortens detection time, etc., which are beneficial in detecting SARS-CoV-2. This review shares recent advances in POCT-based testing for COVID-19 and how it is better suited to help diagnose in response to the ongoing pandemic.

## 1. Introduction

Although considerable methods have been constructed to detect infectious diseases, early-stage screening plays an important role in controlling the spread of viral infections and protecting it from creating a catastrophe. Viruses cause huge numbers of people to suffer from diseases, congenital disabilities, and even death, and they also have the potential to mutate rapidly and spread rapidly through different pathways. Currently, many discoveries are related to understanding various aspects of virology, and viruses remain a major cause of disease [1].

In the 21st century, the severe acute respiratory syndrome coronavirus (SARS-CoV) resulted in significant morbidity and mortality. The coronavirus disease 2019 (COVID-19) caused by the extreme acute SARS-CoV-2 virus was discovered in December 2019 and has spread rapidly around the world [1,2,3]. COVID-19 is the product of SARS-CoV-2 infecting host cells via the angiotensin-converting enzyme 2, which has a higher transmission potential than SARS [4]. World Health Organization has recognized the COVID-19 outbreak as a pandemic. Currently, the primary approach to controlling a pandemic situation is to achieve an accurate diagnosis of the virus to control and break the spread of the virus quickly [5,6]. Besides performing large-scale testing, focusing on isolating infected areas using POCT can provide treatment faster and more accurately, saving lives [7,8]. Rapid devices or biosensors for detecting viruses must be constructed to enable early detection and diagnosis. Generally, the principles of detection strategies for SARS-CoV-2 are usually divided into several categories depending on the biomarkers of analysis: antibodies and nucleic acids. Nucleic acid testing, including gene sequence and reverse transcription-polymerase chain reaction (RT-PCR), is sensitive and stable, and these tests are suitable for large diagnostic in laboratories. On the other hand, the antibodies detected in the blood are immunoglobulin G (IgG) and immunoglobulin M, which can be constructed for the sensitive detection of SARS-CoV-2 [9]. Notably, applying the above strategy RT-PCR for rapid detection in the field outside the laboratory setting is extremely difficult. Due to the rapid evolution of COVID-19 disease and the limited capacity of laboratory-based molecular assays, there is an urgent need for a rapid POCT strategy for the clinical diagnosis of COVID-19 outside the laboratory setting that can provide results within minutes for rapid decisions on patient management. Although many POCT methods are capable of detecting viruses, they use large amounts of reagents, are costly, and have multiple complex spiking sessions that lengthen the detection time and increase human error [10]. These inevitably add to the problems of reliability and sensitivity of the test results.

How do we address the challenges of POCT in SARS-CoV-2 sensitivity and rapidity detection? Microfluidic chips, also known as lab on a chip (LoC), have the characteristics of rapid detection, low cost, and are an ideal tool for simplifying complex small laboratories processes on a tiny device that can integrate sample handling, analysis and other processes and provide the necessary information from a small number of samples [11,12,13,14,15]. Decades of research into microelectronics and microelectromechanical-systems technology have opened up new avenues for developing microfluidic devices to detect viral infections [16,17,18]. Microfluidic chips can be used as a fast, accurate, and automated platform for detecting viral nucleic acids, the number of antibodies in the serum, etc. [1,4]. Combining microfluidic technology with POCT testing can therefore achieve goals that are not possible with conventional biomedical testing, making it easy to diagnose viruses in situ, even in remote locations.

**Figure 1:**
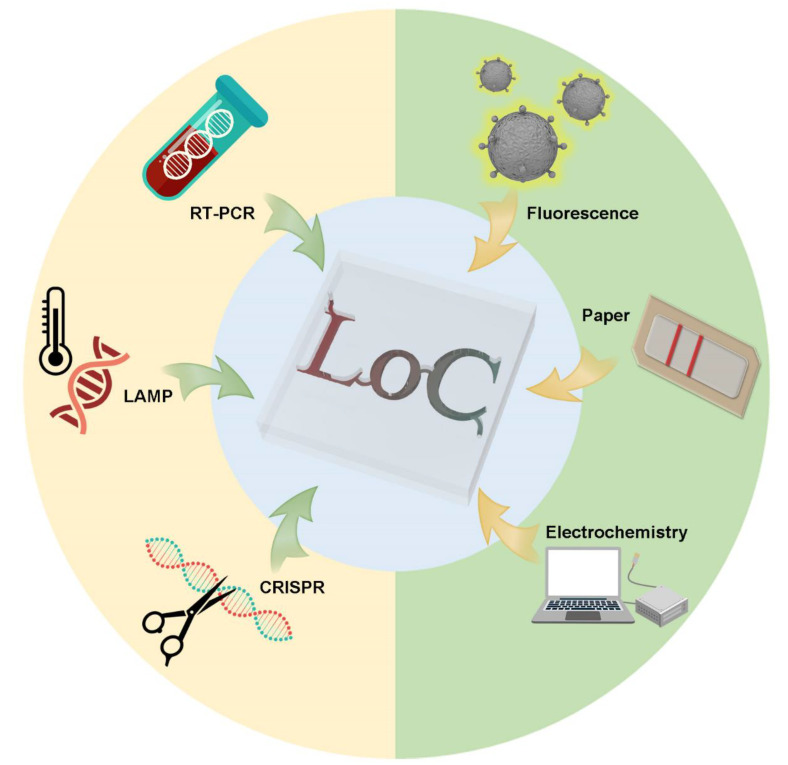
Schematic view of POCT methods for microfluidic use to detect SARS-CoV-2.

Building on the above, we review the existing POCT methods based on the microfluidic technique. We also review some under-development commercial POCT microfluidic methods, which use nucleic acid assays, immunoassays, biosensor assays and other assays for rapid standard diagnostics of COVID-19, shown in Figure 1.

## 2. Nucleic Acid Tests (NAT) Based on Microfluidics

NAT’s high sensitivity and specificity make it the first choice for SARS-CoV-2 detection, especially when COVID-19 antibodies are not yet mass-produced [19,20]. The primary process of nucleic acid detection includes sample collection, virus enrichment, amplification, and detection. However, nucleic acid detection requires multiple rounds of amplification and a long reaction time. Improper primer design and contamination can lead to false negatives and false positives. Microfluidics can control fluid more accurately, transfer heat faster and integrate multiple functions. Therefore, microfluidic technology can provide miniaturization and a high-efficiency solution for nucleic acid detection [21]. Nucleic acid methods based on microfluidics, such as RT-PCR, loop-mediated isothermal amplification (LAMP), and clustered regularly spaced short palindromic repeats (CRISPR), have been used in recent years to identify SARS-CoV-2. This section tells the story and narrates the most recent findings of scientific research teams and marketed POCT equipment.

### 2.1. RT-PCR Tests

RT-PCR combines RNA reverse transcription and complementary DNA (cDNA) PCR technologies. The basic steps for detecting SARS-CoV-2 by RT-PCR can be summarized as follows. Common sample collection forms are oropharyngeal, nasopharyngeal, or anal swabs [22]. The virus is filtered and purified from the sample. After extracting RNA, reverse transcriptase produces cDNA. The target fragment was amplified and synthesized by DNA polymerase using cDNA as a template. Currently, RT-PCR has become the gold standard for COVID-19 diagnosis [23].

Microfluidic chips’ channels have cross-sectional dimensions in micrometers and fluid volumes in microliters [24,25]. On the one hand, the smaller volume significantly reduces reagent consumption and greatly saves costs. High integration of different functions in a small space is also suitable for POCT development requirements [26]. On the other hand, the large surface–volume ratio allows diffusion and heat conduction to be efficient [27,28]. Microfluidics can improve detection accuracy and shorten detection time for RT-PCR using mass and heat transfer methods following basic hydrodynamic rules, which helps with the required repeated temperature changes.

Whole-genome sequencing is essential for virus recognition, PCR primer design, and vaccine development. Rapid sequencing could allow people to respond sooner and cause minor damage as the virus mutates. Li et al. reported an efficient whole-genome sequencing workflow based on microfluidic [29]. They integrated one-step RT-PCR amplification in the microfluidic system and precisely controlled µL fluid in microchannels and microchambers. In the integrated fluidic circuit chip, each sample and primer well contain only 5 µL and 10 µL mixtures, respectively. Although the smaller volume requires more efficient enrichment methods and more genomic copies due to the small amount of RNA in the sample, it effectively avoids tedious operations and cross-contamination between samples.

The extraction and purification of viral RNA is the key to accurate detection by RT-PCR. The participation of microfluidic devices can make this process smaller and faster. Turiello et al. developed an automatable rotationally driven microfluidic platform for enriching and purifying SARS-CoV-2 RNA [30]. The device uses nanotrap magnetic virus particles to isolate the virus for sample enrichment, which removes the complex matrix and avoids inhibiting RNA amplification and detection. The enzyme lysis mixture in the device lyses the virus capsids, releasing the pathogenic nucleic acid and realizing the purification process. Six samples can be preprocessed simultaneously in 15 min and used for RT-PCR. The effectiveness of the device is comparable to that of commercial kits. This automated sample preparation reduces operator involvement, effectively avoiding sample contamination and reducing the risk of infection.

**Figure 2:**
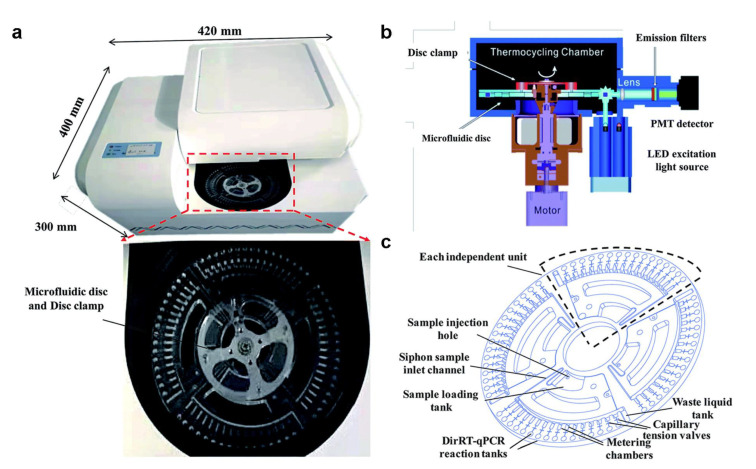
(**a**) Photo of a portable centrifugal microfluidic device. (**b**) Schematic diagram of each component of the microfluidic device. (**c**) Schematic diagram of the structure and functional areas of the centrifugal microfluidic chip. (Reprinted/adapted with permission from Ref. [34]. Copyright 2020 Royal Society of Chemistry).

Microfluidic biochips and portable detection devices using RT-PCR technology provide strong support for the rapid and accurate detection of SARS-CoV-2. Centrifugal microfluidic chips are widely used in disease diagnosis [31,32]. They do not rely on expensive equipment and only use the centrifugal force generated by the rotation of the drive motor to drive the fluid. In addition, the centrifugal microfluidic chip enables highly integrated and automated multiple detections, improving functionality and practicality [33]. Figure 2 shows a direct RT-PCR method developed by Ji et al., based on a centrifugal microfluidic chip system [34]. In addition to the microfluidic chip and the drive motor, the system also contains an optical detection unit and a temperature control unit. The optical detection unit uses the LED Light Source as the excitation source, and a photomultiplier tube with emission filters to collect the fluorescence. This system can detect and classify SARS-CoV-2 and influenza A and B in 1.5 h without professional operators. The centrifugal microfluidic is commonly used for nucleic acid detection. It is simple to drive, does not need an external pump to control the fluid, and has almost no possibility of polluting the environment. In addition, the microfluidic chip has the advantage of realizing multiple target detection. Distinguishing COVID-19 and influenza, which have similar symptoms, could provide access to mass early screening and reduce medical pressure [35,36,37,38].

The disadvantages of RT-PCR cannot be ignored either. Due to the virus incubation period and differences in the amount of virus carried by individuals, upper respiratory tract sampling is often unable to obtain sufficient virus load samples the first time [39]. In addition, irregular operation of sampling personnel and contamination of samples may lead to inaccurate test results [40]. These problems limit the use of RT-PCR. We believe that the standardization and automation of the sampling process in microfluidic chips may improve this problem.

### 2.2. LAMP Tests

**Figure 3:**
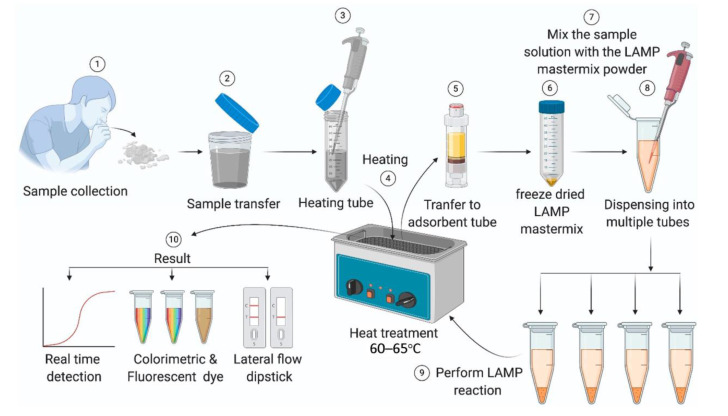
Schematic diagram of the LAMP detection process [45].

Isothermal amplification technology (IAT) can overcome the limitation of multiple thermal cycles of traditional PCR and carry out nucleic acid amplification under constant temperature. The development of IAT has greatly simplified PCR analysis steps, reduced detection time, and reduced operational difficulty [41,42,43,44]. LAMP is vital to IAT and can detect multiple targets simultaneously in a single reaction. Some studies have shown that LAMP is more sensitive and accurate than RT-PCR in detecting SARS-CoV-2. Figure 3 presents the reaction process of LAMP [45], which can be summarized as amplifying LAMP-specific primers and extracted viral nucleic acids at a constant temperature of 60–65 ℃. The results are usually produced in colorimetry or fluorescence and read out by naked eyes or a small device. LAMP does not rely on large instruments in the detection and reading process. It is suitable for self-screening in communities and families.

**Figure 4:**
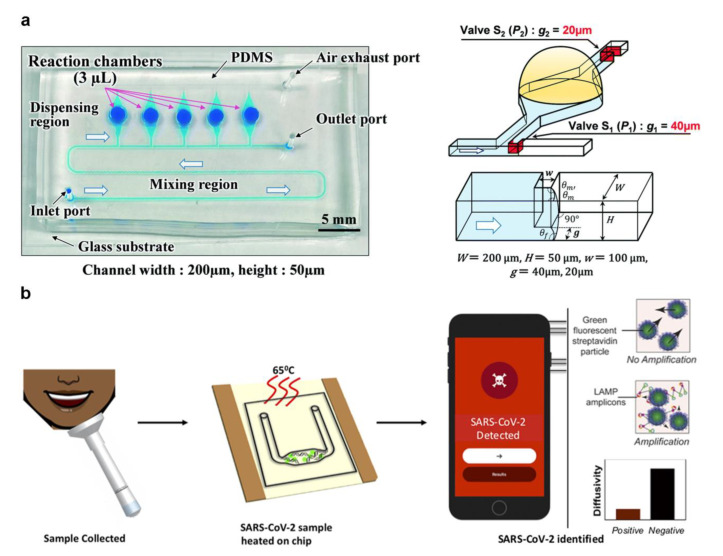
(**a**) A microfluidic chip structure of sequential fluid dispensing. (Reprinted/adapted with permission from Ref. [46]. Copyright 2021 Royal Society of Chemistry). (**b**) A microfluidic chip integrated LAMP and particle diffusometry. (Reprinted/adapted with permission from Ref. [49]. Copyright 2022 Elsevier).

Natsuhara et al. designed a microfluidic chip with mixing and dispensing regions that can dispense continuous fluid (Figure 4a) [46]. In each chamber, infectious diseases such as COVID-19 and influenza can be analyzed by LAMP-based colorimetry. Changing the microchannel structure causes different cut-off pressures to achieve passive fluid dispensing. Lyu et al. developed a droplet array slip-chip that can be used for high-throughput detection of COVID-19 [47]. The fluid movement by sliding the chip is a simple step that avoids the precise bonding problems of traditional chips with high-precision microchannels. This design has tunability potential. This could allow the customizable design ability based on the modification of the size and density of the chamber while improving the detection efficiency. de Oliveira et al. reported a centrifuge microfluidic device manually controlled using a fidget spinner for LAMP-based SARS-CoV-2 detection [48]. The device is device-independent and portable, but the speed control is inaccurate, and improper operation may cause errors. The above works focus on designing a microfluidic chip structure to control the fluid, but there is a lack of a portable signal readout device.

Colorimetric and fluorescence methods are the most common readout method for LAMP detection results acquisition. The smartphone has become a reliable signal readout device for LAMP with enhanced image function and computing capability. Colbert et al. developed a SARS-CoV-2 detection technique by combining LAMP and particle diffusometry (Figure 4b) [49]. The samples containing fluorescent beads post-RT-LAMP can be imaged with the smartphone. There was a negative correlation between diffusion coefficient and virus content in the process of particle amplification. The virus can be detected in 35 min by using a smartphone to monitor the fluorescent signal. Lim et al. reported a highly integrated cartridge for SARS-CoV-2 detection and classification of different variants [50]. The device comprises an injection port, a micromixer, a detection area, a fluorescent excitation light source, and a pull-out chip placement area. The all-in-one portable device uses a smartphone to read signals and develops a unique program to analyze the detection signals and results, realizing complete analysis automation. The lack of reliance on specialized equipment allows LAMP-based microfluidic systems to provide POCT strategies for more ordinary people.

LAMP integration into the microfluidic system further reduces volume and operational requirements, making it a reliable way for people to accurately and rapidly self-diagnose COVID-19. However, LAMP requires the design of complex specific primers, which results in some diseases that cannot be detected by LAMP. In the face of rapidly mutating SARS-CoV-2, primer design will slow detection technology development [51]. Moreover, LAMP could not distinguish nonspecific amplification products caused by aerosol pollution, impurities in the sample base, enclosed environment destruction, and other factors. It may make highly sensitive LAMP produce false positives [52,53]. How to improve the system’s airtightness to avoid contamination of reagents is a problem that the designer needs to consider. For microfluidic systems, the injection process needs to be designed to be airtight and fluid-driven to avoid the entry of external impurities.

### 2.3. CRISPR-Associated Proteins System (Cas) Tests

CRISPR, dubbed “molecular scissors”, is a powerful gene-editing technology [54]. It originates from the bacteria’s acquired immune system, which cuts the DNA sequence of the invader with the intervention of CRISPR RNA (crRNA) and Cas enzymes [55]. CRISPR has two main classes. Class 1 interference complexes contain CRISPR-I, III, and IV, which employ a multieffector complex to cleave the target genome sequence using crRNA [56]. Class 2 is the system consisting of CRISPR-II, V, and VI, which uses a single multidomain Cas protein to interfere with the target region [57,58]. Cas12 and Cas13 in Class 2 are more suitable for SARS-CoV-2 detection among CRISPR-related proteins.

**Figure 5:**
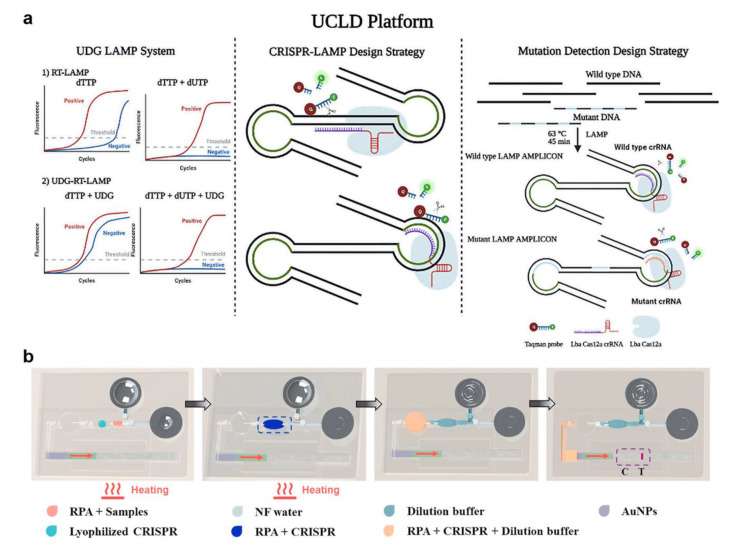
(**a**) Overview of the universally stable and precise CRISPR-LAMP detection platform. (Reprinted/adapted with permission from Ref. [59]. Copyright 2020 American Chemical Society). (**b**) A lateral flow microfluidic device that does not rely on external devices and has a hand-warmer pouch as its power source. (Reprinted/adapted with permission from Ref. [62]. Copyright 2022 Elsevier).

Zhang et al. constructed a CRISPR system combined with fluorophore-quencher DNA probes (Figure 5a) [59], which was a means of signal amplification and readout. Ramachandran et al. used isotachophoresis (ITP) to obtain an electric-field gradient on a microfluidic chip, combined with LAMP and CRISPR, to diagnose COVID-19 in 35 min [60]. In this system, Cas12 is combined with the guide DNA, the complex binds specifically to the target DNA, and the fluorophore-quencher DNA probes are cleaved. ITP optimizes DNA purification and speeds up the reaction in this process. CRISPR is greatly limited by the time-consuming nucleic acid amplification and the reliance on large instruments to read fluorescent signals. Silva et al. developed a catalase-mediated assay for detecting SARS-CoV-2 based on CRISPR [61]. Ribonucleoprotein complex was used to detect a SARS-CoV-2 reverse-transcribed DNA/RNA heteroduplex target. The bubble signal was produced by the transcleavage collateral activity of the Cas12a protein on a Catalase:ssDNA probe. The assay does not require nucleic acid amplification and can be read by smartphone photographing bubble signals. However, this analysis method can only be used for qualitative rather than quantitative detection. Some researchers have developed testing methods that are entirely independent of external devices. Li et al. reported a lateral flow microfluidic device using a hand-warmer pouch as a heat source (Figure 5b) [62]. They prestore the freeze-dried powder of the reagent in a reaction chamber. The user can push the liquid with fingers and read the results with the naked eye. The simple and device-independent operation will be the trend of SARS-CoV-2 detection technology.

As an emerging technology, CRISPR-based detection takes less time and does not rely on complex equipment but is conducive to the quality of the equipment being used. In addition, in the face of the accelerated mutation rate of SARS-CoV-2, CRISPR can still perform highly sensitive detection for different mutation types. In addition, CRISPR can also detect results sensitively in samples with low viral loads [63,64]. However, the off-target effect of CRISPR can seriously affect the specificity of detection results, and the design complexity of crDNA also severely limits the development of this technology [65].

### 2.4. Commercial NAT Microfluidic Implementation

NAT for diagnosing COVID-19 has become a very reliable method for diagnosis [66]. Many commercial products used in Table 1 further classify these NAT devices. ID NOW™ COVID-19 from Abbott Diagnostics Scarborough Inc takes 15 min per test and targets the RNA-dependent RNA polymerase gene using an isothermal system. VitaPCR™ SARS-CoV-2 Gen2 Assay of Credo Diagnostics Biomedical Pte. Ltd. performs real-time PCR targeting N1, N2-Nucleocapsid Gene. Each test takes 20 min to finish. The device shows a positive (90.0%) and negative predictive value (99.0%) with high sensitivity (94.6%) and specificity (98.1%). The BioFire^®^ Respiratory 2.1-EZ from BioFire Diagnostics, LLC can run simultaneous detection, which provides a wider range of diagnosis options for infectious diseases besides COVID-19. The nested multiplex PCR detection is advantageous, especially in dealing with different strains of COVID-19 at the same time. The Spike protein (S) gene and Membrane protein (M) gene are the targets for this device. 1copy COVID-19 qPCR Kit of 1 drop Inc focuses on RT PCR and targets. E gene is for beta coronavirus and the RdRp gene is for SARS-CoV-2. BIOSYNEX COVID-19 BSS gives an edge over only plasma or serum-dependent methods. Whole blood, serum, or plasma can all be used for detection. The kit also has a longer shelf time of 18 months. AQ-TOP COVID-9 Rapid Detection Kit PLUS of SEASUN BIOMATERIALS uses real-time Hyper RT-PCR, taking 30 min to produce results. The targets are SARS-CoV-2 Orf1ab and the human RNase P gene for this. Impressively, it has a claim to have no false positive due to its gene sequencing specific to the PNA probe. Novel Coronavirus (2019-nCoV) RT-PCR Detection Kit (Lyophilized) from Shanghai Chuangkun Bitech Inc. detects SARS-CoV-2 RNA using nasopharyngeal swabs, oropharyngeal swabs, sputum, and alveolar lavage fluid specimens. The kit targets the ORF1ab gene and N gene of SARS-CoV-2 with the real-time reverse transcription-polymerase chain reaction method.

## 3. Microfluidics-Based POCT on Immunoassay

### 3.1. Fluorescence-Assisted Tests

Fluorescent elements to identify COVID-19 targeting the nanoparticles provides features such as large target surfaces, which contribute to the rapid identification of viral infections and play an important role in the COVID-19 detection system [76,77,78]. Nanoparticles can influence target elements [79,80] and amplification or sequencing steps [81,82], giving a better control with the overall detection methodology.

**Figure 6:**
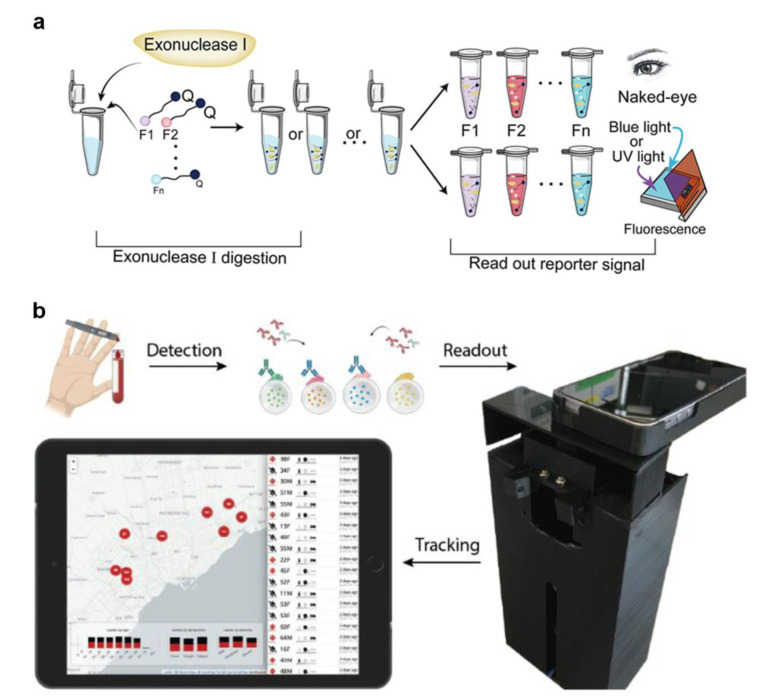
(**a**) Screening and optimization of the ssDNA-FQ reporters for RAVI-CRISPR assays. (Reprinted/adapted with permission from Ref. [91]. Copyright 2022 American Chemical Society). (**b**) Schematic for the smartphone antibody detection workflow. (Reprinted/adapted with permission from Ref. [92]. Copyright 2021 American Chemical Society).

Due to the readability of fluorescence, it is the most prevalent signal-output nanoparticle. Fluorescence imaging has versatility and can be applied to various materials, including paper, polymers, and unstructured materials. [83,84,85]. It can also be used with smartphones or desktop scanners for automated analysis [79,86]. Virus avian influenza genomic DNA, for example, the development of a microfluidic system, was set up with a computer to amplify the target DNA, a bluish light-emitting diode, and a narrowband filter. The fluorescent signals provided by different dyes (EvaGreen, SYBR Green, and FAM fluorescent probe) can be recorded in real time using a smartphone. Ma et al. proposed another method for smartphone viral detection [87]. The group developed the process, which took 40 min to detect the H1N1 virus. Colorimetric methods that include modifying surfaces with chemical components may also be used to alert the consumer to the presence of viruses [88,89,90]. Their system used colorimetric LAMP with a passive microfluidic unit and an optical detector while regulating and analyzing the results using a smartphone. This makes developing POCT devices easier [72,76] and has accurate detection compatibility compared to the laboratory [79]. Xie et al. established a RApid VIsual CRISPR (RAVI-CRISPR) that can fabricate an instrument-less colorimetric POCT to detect SARS-CoV-2 [91]. In detail, exonuclease I cleavage (left) and subsequent inspection under blue or UV light or by the naked eye of colorimetric changes in the reaction solution (right) are used to determine ssDNA-FQ reporter activity (Figure 6a). In addition, a smartphone imaging platform loaded with software can diagnose SARS-CoV-2 at different sampling dates and infectious severity (Figure 6b) [92]. They fabricated a fluorescent-material (quantum dot) barcode device with about 3 times greater clinical sensitivity than lateral flow assays. In addition to this feature, the developed platform has the potential to realize antibody detection, immunity surveillance, and vaccination-induced seroconversion monitoring.

### 3.2. Paper-Based Tests

**Figure 7:**
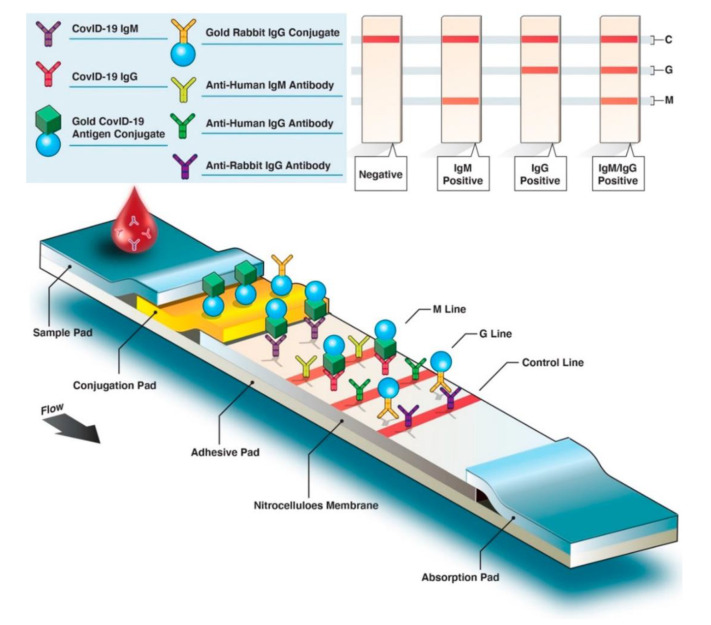
Structure and flow diagram of colorimetric lateral flow immunoassay. (Reprinted/adapted with permission from Ref. [102]).

The advantage of the lateral flow assay (LFA) is that the color changes caused by the accumulation of gold nanoparticles can be read by naked eyes, providing a convenient way for large-scale screening of diseases [93,94,95,96]. With the development of nanotechnology, materials such as carbon nanoparticles and carbon nanotubes have significantly improved the sensitivity of LFA [97]. Researchers are also developing more sensitive technologies based on LFA that are not read by the naked eye [98]. Typically, patient antibodies (IgG, IgM, or IgA) produced against a specific SARS-CoV-2 antigen can be detected by LFA. Gold nanoparticle-labeled conjugated SARS-CoV-2-specific antigens bind with host antibodies. As antibody–antigen complexes migrate through the membrane, bound anti-SARS-CoV-2 IgM antibodies interact with anti-IgM secondary antibodies on the M line and anti-SARS-CoV-2 with anti-IgG on the G line. If the blood sample lacks SARS-CoV-2-specific antibodies, just the control (C) line will be visible (Figure 7). To create a commercial-grade LFA for detecting SARS-CoV-2 antigen, Benjamin D. Grant et al. employed publicly available reagents and a half-strip test procedure. The detection limit for recombinant antigen is 0.65 ng/mL [99]. Wen and Huang et al. optimized the pH, antigen concentration, and other parameters to ensure lateral flow immunoassay strip stability. The results for IgG and IgM antibodies against SARS-CoV-2 were both specific [97,100]. The specificities and sensitivities of IgM detection were 100% and 93.3%, respectively. Chen et al. used a fluorescent reporter lanthanide-doped polystyrene nanoparticles to detect anti-SARS-CoV-2 IgG in clinical samples [101]. The commercialization of the LFA approach for diagnosing COVID-19 has been widespread, and it can be implemented with simple equipment and low requirements for the detection environment. Additionally, it is capable of on-the-go inspection and has increased applications [102].

However, the lack of processing before testing can lead to some compounds skewing results and leading to false-positive results. To lower the number of false positives, improving the strip materials’ filtering capabilities is required by optimizing the strip materials’ processing, changing reagent compositions, and improving the anti-interference abilities. Yu et al. used an NLFA to detect three SARS-CoV-2 virus genes simultaneously: RdRp, ORF3a, and the N protein gene [103]. Before the NLFA method, amplification utilizing RT-PCR or another approach was required. NFLIAs have also been investigated for the detection of SARS-CoV-2. Several LFIAs have been developed to detect antibodies in the blood of COVID-19 patients due to the SARS-CoV-2 virus. Focusing on antibody detection may result in false-negative testing when the disease is in its early stages. This is because antibodies may be at undetectable levels in the days following infection [104,105]. During the COVID-19 epidemic, lateral flow technologies were created that are portable, fast-acting, low-cost, and simple to use. They are quickly becoming one of the best approaches for performing POC testing. This section has presented an overview of lateral flow assay testing improvements and how their sensitivity and specificity might be enhanced.

### 3.3. Electrochemical Biosensors

While electrochemical biosensors are tiny devices that are affordable and easy to shrink, they are particularly popular due to their simplicity and lower cost. In an electrochemical biosensor, real-time, specific, and accurate target monitoring generally relies on a tailored electrode, which acts as either the receptor or transducer depending on the need [106,107,108]. Potentiometric or amperometric signals, related to the presence of the analyte of interest, can be extracted from the sensing electrode’s information [109,110,111].

**Figure 8:**
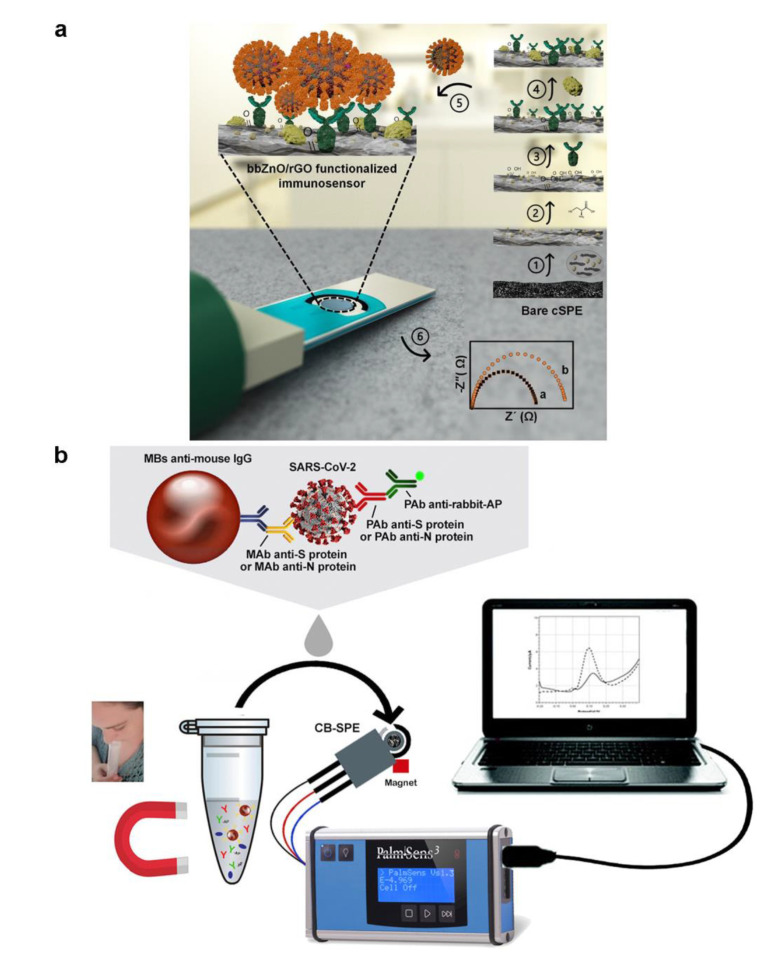
(**a**) Graphical illustration of the step-by-step immuno-biosensor preparation. (Reprinted/adapted with permission from Ref. [113]. Copyright 2020 American Chemical Society). (**b**) Schematic diagram of electrochemical biosensor detection of a saliva sample. (Reprinted/adapted with permission from Ref. [115]. Copyright 2020 Elsevier).

A portable electrochemical analysis device, combining surface immobilization with smartphone software, can acquire data rapidly and potentially be a smart POCT tool to help decrease the need for cumbersome gear [112]. The ability to identify viruses is even further enhanced by the ongoing research exploring various electrochemical solutions and, perhaps, cuts the cost and amount of time necessary to operate. Sanati-Nezhad et al. developed an electrochemical immuno-biosensing for the detection of SARS-CoV-2 nucleocapsid protein antigens by bbZnO/rGO nanocomposite coated on carbon screen-printed electrodes, which can improve the adsorbing of antibodies (Figure 8a) [113]. The fabricated device was then linked to the readout system, generating the resulting electrochemical signals in the presence of the COVID-19. The electrochemical immuno-biosensor could yield acceptable sensitivity and LOD, which was profoundly significant in early COVID-19 diagnosis. Ali et al. constructed a three-dimensional reduced-graphene-oxide electrode and connected it with a microfluidic device as an electrochemical sensor by introducing improved 3D-printing technology [114]. To allow for the detection of antibodies specific to SARS-CoV-2, viral antigens were attached to 3D electrodes, resulting in a detection limit of 2.8 × 10^−15^ M. Fabiani et al. created a miniaturized electrochemical sensor for the detection of SARS-CoV-2 using magnetic beads with a carbon black-based electrode (Figure 8b) [115]. Magnetic beads can increase preconcentration and minimize washing while retaining sensitivity and reliability. The external magnetic field can also help remove seasonal H1N1 influenza virus interference detection.

Two recent studies have used paper’s low-cost, mobile, and disposable features as an electrochemical sensor substrate. To detect SARS-CoV-2 antibodies in about thirty minutes, Yakoh et al. designed an electrochemical apparatus that prints electrode patterns on paper [116]. The paper-based sensor has great potential as a POCT platform because of its unique, inexpensive, portable, and disposable qualities. The paper-based electrochemical substrate was utilized to attach the sensing probes, which were employed to develop a device that could detect nucleic acids [117]. Sensing materials, such as gold nanoparticles, are precisely designed to maximize the sensor’s sensitivity and output signals within five minutes. It is additionally able to provide a quantitative range for the detection of SARS-CoV-2 targets, from 585.4 copies/μL to 5.854 × 10^7^ copies/μL, with a sensitivity of 231 copies/μL, which allows for the tracking of the infection progression in suspected cases. Data transmission to end-user devices is another crucial electrochemical-sensing component. Zhao et al. demonstrated that a smartphone’s electrochemical sensor could detect SARS-CoV-2 RNA, relieving the need for large-scale equipment and lab processes [118]. Portable testing may be possible if this “plug-and-play” setup is available to clients. A new study further integrated the electrochemical platform with a wireless module to help develop graphene electrodes that allow ultrarapid COVID-19 identification [119]. ‘SARS-CoV-2 RapidPlex′ can detect SARS-CoV infection while being inexpensive and highly sensitive, providing information on the three important components of COVID-19 disease (viral infection, immunological response, and disease severity) with the potential to be conducted at home.

### 3.4. Commercial Microfluidics Immunoassay Products

Many commercialized POCT devices are available based on immunoassay technology (Table 2) [120]. A. Menarini Diagnostics, Italy, developed immunoassay-based ANTIGEN RAPID TEST CASSETTE SARS-CoV-2 (SWAB). This takes a nasopharyngeal swab and uses nucleocapside protein. The overall process takes about 15 min. AAZ-LMB, France, uses the ELISA in their COV-QUANTO with plasma sampling. Nevertheless, this takes a long time. The process can take up to 210 min to finish. Panbio COVID-19 Ag Rapid Test by Abbott Rapid Diagnostics, Switzerland, uses the near-POC method, which takes 15 min to perform the rapid test from a nasal swab or nasopharyngeal swab. The accuracy this device claims to have 100% accuracy. Similarly, AccuBioTech Co., Ltd., China, launched ACCU-TELL^®^ SARS-CoV-2 Neutralizing Antibody Cassette with near POC, which takes 10 min to finish detection from whole blood. Another POC device, Flowflex SARS-CoV-2 Antigen Rapid Test from Acon Biotech (Hangzhou) Co., Ltd., China, uses nucleocapsid protein as a detection principle. However, detection accuracy varies with the sampling, with 98.7% for the nasopharyngeal swab and 98.8% for the nasal swab. Actim Oy, Finland, uses immunochromatography in their Actim SARS-CoV-2 device to detect SARS-CoV-2 taking 15 min from sampling to result. InfectCheck^®^-COVID-19 IgG/IgM Test of Affimedix, Inc., United States and 2019-nCoV Antigen Device (Anterior Nasal Swab) AMS UK (NI) Ltd., United Kingdom uses similar technology, immunochromatography, but differs in sampling. Both the devices have good accuracy, with 99.8% and 98.1%, respectively.

## 4. Conclusions and Perspectives

This review briefly discusses why microfluidic can be used as a POCT strategy for detecting SARS-CoV-2. Microfluidic technology can integrate complex analytical processes in small volumes, enabling sampling, mixing, separation, enrichment, washing, temperature control, and other operations. It can provide a rapid, accurate, and automated platform for SARS-CoV-2 detection at a low cost. Researchers have integrated typical NAT applications into microfluidic chips, including PT-PCR, LAMP, and CRISPR. In addition, highly sensitive analysis methods represented by fluorescence-assisted tests, paper-based tests, and electrochemical biosensors have also been widely used. False negatives may result from samples with low viral loads, which may also be caused by external environmental contamination and nonspecific reactions. While sensitivity and specificity have improved, occasional errors can delay prevention and treatment. Although the performance of these devices has been impressive, there is still a long way to go from leading-edge results in the laboratory. The operational requirements of commercial equipment make test results less reliable, and the product is not affordable for most people. We believe that as testing needs continue to increase, more exemplary methods can be introduced to the market.

As COVID-19 spreads and mutates, rapid, accurate testing in communities and households is critical. We believe that future detection methods can be improved in the following aspects. First, the development of standardized and automated sampling procedures to avoid detection errors caused by incorrect sampling and to reduce the burden on human resources. Second, it is necessary to reduce the difficulty of the operation and the detection time, which requires the complete coordination of processes such as reagent preparation, instrumentation, test procedures, and signal analysis. A simple-enough preparation process is fundamental to meeting the testing needs of developing regions. The premise of the large-scale popularization of technology is how to enable ordinary people to self-test rapidly. The third is the accuracy of the test results. Many tests are not designed to produce false negatives for samples with low viral loads and may also be caused by external environmental contamination and nonspecific reactions. While sensitivity and specificity have improved, occasional errors can delay prevention and treatment.

## Figures and Tables

**Table 1:** Some commercialized POCT devices are based on NAT.

Product Name	Manufacturer	Method	Time (min/test)	Target	Sample Type	Sensitivity ^1^/Specificity ^2^	Storage and Stability	Limit of Detection	Reference
ID NOW™ COVID-19	Abbott Diagnostics Scarborough, Inc.	NEAR	15	COVID-19 RdRp gene	Nasal, Throat, and Nasopharyngeal swabs	93.3%/98.4%	2–30 °C	125 genome equivalents per mL	[67]
VitaPCR™ SARS-CoV-2 Gen2 Assay	Credo Diagnostics Biomedical Pte. Ltd.	Real-Time RT-PCR	20	N1, N2-Nucleocapsid Gene	Nasopharyngeal and Oropharyngeal swabs	100%/100%	+2 °C/+8 °C	30 copies/reaction	[68]
The BioFire^®^ Respiratory 2.1-EZ (RP2.1- EZ) Panel (EUA)	BioFire Diagnostics, LLC	Nested multiplex PCR	45	Spike protein gene, Membrane protein gene	Nasopharyngeal swabs	97.1%/99.3%	15–25 °C	500 copies/L	[69]
1copy COVID-19 qPCR Kit	1drop Inc	RT-PCR	22	E gene for beta coronavirus and the RdRp gene for SARS-CoV-2	Nasopharyngeal and Oropharyngeal swabs	-/-	>20 °C	200 copies/mL	[70]
Biosynex COVID-19 Ag+ BSS Rapid Test	BIOSYNEX S.A., Switzerland	RT-PCR	10	N-protein detection	Nasopharyngeal swab	97.5%/100%	2–8 °C	750 TCID50/mL	[71]
Foaming Test	PharmaNona	POC/Near POC	1	Nucleic acid	Urine	92%/89%	-	-	[72]
AQ-TOP COVID-9 Rapid Detection Kit PLUS	SEASUN BIOMATERIALS	real-time Hyper RT-PCR	30	SARS-CoV-2 Orf1ab and the human RNase P gene	Bronchoalveolar lavage, Mid-turbinate swab, Nasal swab, Nasopharyngeal swab, Oropharyngeal swab, Sputum	-/100%	−20 °C	1 copy/uL in single reaction	[73]
Novel Coronavirus (2019-nCoV) RT-PCR Detection Kit (Lyophilized)	Shanghai Chuangkun Bitech Inc.	RT-PCR	70	COVID-19 S gene	Nasopharyngeal swab	95%/100%	-	500 copies/uL	[74]
SARS-CoV-2 IgM/IgG Antibody Assay Kit	Zybio Inc	Colloidal Gold method	15		Serum, Plasma, Whole Blood	IgM 98.67%, IgG 95%/IgM 91.11%, IgG 95%	4–30 °C	Not Applicable	[75]

^1^ Sensitivity is the proportion of samples that are actually positive that are judged to be positive and is calculated as the ratio of true positives divided by true positives + false negatives. ^2^ Specificity is the proportion of samples that are actually negative that are judged to be negative and is calculated as the ratio of true negatives divided by true negatives + false positives.

**Table 2:** Some Commercialized POCT devices based on immunoassay.

Product Name	Manufacturer	Method	Time (min)	Sampling	Accuracy ^1^	Sensitivity ^2^	Specificity ^3^	Detection Principle	Reference
ANTIGEN RAPID TEST CASSETTE SARS-CoV-2 (SWAB)	A. Menarini Diagnostics, Italy	Immunoassay	15	Nasopharyngeal swab	98.74% (95%CI: 96.80–99.66%)	96.72% (95%CI: 88.65–99.60%)	99.22% (95%CI: 97.21–99.91%)	Nucleocapside protein	[121]
COV-QUANTO	AAZ-LMB, France	ELISA	210	Plasma, serum	85% (CI: range: 85–115 (Antigen))	93%	98.4%	Immuno-Antigen	[122]
Panbio COVID-19 Ag Rapid Test	Abbott Rapid Diagnostic, Switzerland	Near-POC	15	Nasal swab, nasopharyngeal swab	100%	98%	99%	ImmunoAssay-Antigen	[123]
ACCU-TELL^®^ SARS-CoV-2 Neutralizing Antibody Cassette	AccuBioTech Co., Ltd., China	Near-POC	10	Serum, whole blood	100% (Neutralizing antibody)	100% (Neutralizing antibody)	100% (Neutralizing antibody)	Immuno-Antibody	[124]
Flowflex SARS-CoV-2 Antigen Rapid Test	Acon Biotech (Hangzhou) Co., Ltd., China	Lab-based, near-POC/POC	15	Nasal swab, nasopharyngeal swab	98.7% (Nasopharyngeal swab) 98.8% (nasal swab)	97.1% (Nasal swab) 97.6% (nasopharyngeal Swab)	99.5% (Nasal swab) 99.4% (nasopharyngeal Swab)	Nucleocapsid protein	[124]
Actim SARS-CoV-2	Actim Oy, Finland	Immunochromatography	15	Nasopharyngeal swab	98% (Ct values < 33)	100 (Ct values < 25), 98% (Ct values < 30). 96% (Ct values < 33), 88% (Ct values 9.3–39.8)	100% (Antigen)	Immuno-Antigen	[125]
InfectCheck^®^-COVID-19 IgG/IgM Test	Affimedix, Inc., United States	Immunochromatography	15	Plasma, serum, whole blood	99.8%	98.3%	99.7%	Immuno-Antibody	[126]
2019-nCoV Antigen Device (Anterior Nasal Swab)	AMS UK (NI) Ltd., United Kingdom	Immunochromatography		Anterior nasal swab	98.1%	96.3%	99.5%	Nucleocapsid protein	[127]

^1^ Accuracy is expressed as the sum of the number of true positives and true negatives as a percentage of the number of subjects. ^2^ Sensitivity is the proportion of samples that are actually positive that are judged to be positive and is calculated as the ratio of true positives divided by true positives + false negatives. ^3^ Specificity is the proportion of samples that are actually negative that are judged to be negative and is calculated as the ratio of true negatives divided by true negatives + false positives.

## Abbreviation

Point-of-care testing (POCT); severe acute respiratory syndrome coronavirus (SARS-CoV); coronavirus disease 2019 (COVID-19); reverse transcription-polymerase chain reaction (RT-PCR); immunoglobulin G (IgG); lab on a chip (LoC); loop-mediated isothermal amplification (LAMP); clustered regularly spaced short palindromic repeats (CRISPR); complementary DNA (cDNA); isothermal amplification technology (IAT); CRISPR-associated proteins system (Cas); CRISPR RNA (crRNA); isotachophoresis (ITP); RApid VIsual CRISPR (RAVI-CRISPR); lateral flow assay (LFA).
